# The Spatio-Temporal Distribution and Population Dynamics of Chub Mackerel (*Scomber japonicus*) in the High Seas of the Northwest Pacific Ocean

**DOI:** 10.3390/ani15081135

**Published:** 2025-04-15

**Authors:** Guoqing Zhao, Heng Zhang, Fenghua Tang

**Affiliations:** 1 East China Sea Fisheries Research Institute, Chinese Academy of Fishery Sciences, Shanghai 200090, China; zgq617717@163.com (G.Z.); zhangziqian0601@163.com (H.Z.); 2 Key Laboratory of Polar Ecosystem and Climate Change, Shanghai Jiao Tong University, Ministry of Education, Shanghai 200030, China

**Keywords:** biological characteristics, *Scomber japonicus*, stock assessment, LBB, fishing ground, Northwest Pacific Ocean

## Abstract

Chub mackerel (*Scomber japonicus*) is a typical small pelagic fish in the high seas of the Northwest Pacific Ocean (NPO), with great ecological and economic values. It is crucial to understand the spatio-temporal distribution and the population dynamics of chub mackerel for commercial fishing and fishery management. In this study, we analyzed the population dynamics and stock status of chub mackerel based on commercial fishing data. Results showed that from 2016 to 2021, high catches of chub mackerel were concentrated along the outer edge of Japan or Russia’s exclusive economic zone line, and the gravity center of the fishing grounds moved to the northwest gradually, with a decrease in the average value of catch per unit effort (CPUE) year by year. The fishing pressure may not be the major reason for the decrease in chub mackerel as the resource biomass was at a high level. The body length structure was reasonable, and large individuals were still present. The study highlights the fact that the population status of chub mackerel was healthy in 2016–2021 according to our results. However, it has to be emphasized that resource exploitation and fishery management also need to take into account the influence of climatic conditions.

## Simple Summary

Chub mackerel (*Scomber japonicus)* is the main fishing species in the high seas of North Pacific Ocean (NPO) fishery, bringing great ecological and economic values. In recent years, the catch of chub mackerel has changed somewhat in response to the warming climate, changes in fishing effort, and the implementation of management systems. To provide more useful information for fishery management, we studied the characteristics of the fishing grounds of chub mackerel and evaluated their status of growth and exploitation. We emphasized the importance of climate warming to the dynamics of the chub mackerel population. This study is of great significance to the formulation of management measures for chub mackerel in the high seas of the NPO.

## 1. Introduction

**Figure 1:**
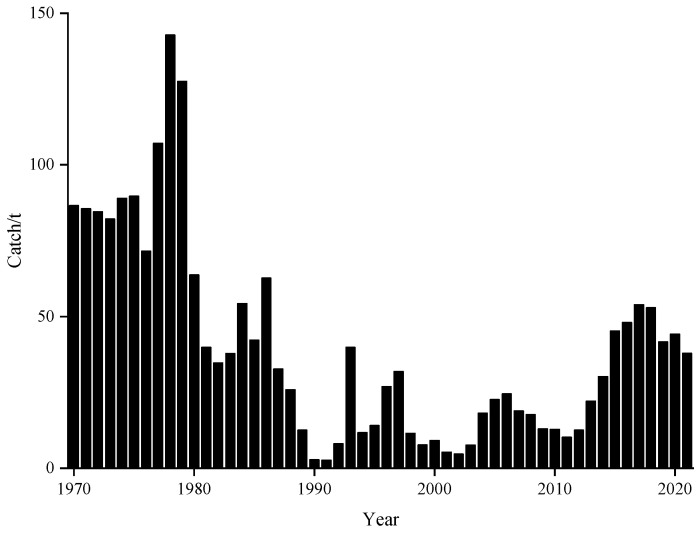
The yearly catch of chub mackerel in the NPO from 1970 to 2021.

Chub mackerel (*Scomber japonicus*) is a typical small pelagic fish with great economic and ecological value, widely distributed along the continental shelf in the adjacent waters from 0 to 300 m in the Pacific, Atlantic, and Indian Oceans, spanning the temperate and subtropical zones [1,2,3], with characteristics of phototropism, vertical movement, and long-distance migration [4]. The Northwest Pacific Ocean (NPO) is the main fishing ground of chub mackerel. According to the results of the latest report of the World Food and Agriculture Organization (FAO), the annual world production of chub mackerel has exceeded 1.3 million tons in the last five years [5], and the production of the NPO accounts for more than 30% of the annual world production in each year [6]. Japan has the longest history of fishing in this area, and other countries fishing in this area including China and Russia [7,8]. Since the 1970s, both the resource and catches of chub mackerel in this area have experienced significant fluctuations. According to the Fisheries Agency of Japan (http://abchan.fra.go.jp/ accessed on 3 May 2023), although the catches have increased significantly in the last decade (Figure 1), the proportion of the resource is still at a historically low level, suggesting that this species has certain exploitation potential [6]. Chub mackerel has been listed as a priority management species by the North Pacific Fisheries Commission since 2015 because of its huge resource and significant commercial value [9]. Current studies on chub mackerel have focused on large-scale analyses using catch statistics, such as fishing ground variability [8], habitat suitability analysis [10], and climate change effects [3,11]. However, the growth and exploitation status of chub mackerel in the NPO, especially in the high seas of the NPO, have not been well described due to the lack of high-quality fishery data from long time series.

In fact, the vast majority of fisheries in our world are not properly managed. According to the FAO reports [5,12], only about 12% of fisheries are well managed nowadays using complex stock assessment models based on the purpose of fisheries management. Fishery stock assessment involves complex scientific analysis, which can provide key suggestions for fisheries management, such as the implementation of a fishing quota system [13]. High-quality fishery stock assessment include three types of data: catches and fishing effort data, abundance indices, and biological data [13]. However, most fishery stocks lack sufficient information for modeling due to the high cost of data acquisition and collection in surveys, making it difficult to use for traditional assessment models [14,15,16,17]. As a result, the “lack of data methods” was highlighted as one of the four main themes at the 2013 World Conference on Fisheries Resource Assessment Methods in Boston [16,18]. Due to their short life cycle, long migration routes, and high vulnerability to large-scale climatic and regional environmental changes [19], chub mackerel fishery is a typical data-poor fishery in the high seas of NPO [20]. At present, some scientists have focused on the population evaluation of chub mackerel in the high seas of NPO. Shi et al. [20] used an length-based Bayesian evaluation (LBB) model to analyze the populations of Chub mackerel, Pacific saury (*Cololabis saira*), and Pacific sardine (*Sardinops sagax*) and concluded that all three fish species were in a relatively healthy population state. Wu et al. [21] explored the influence of environmental factors on the abundance of chub mackerel resources based on a generalized additive model and concluded that the models of Arctic Oscillation Index, sea surface temperature, and sea surface height could better predict their abundance. Ryuki et al. [6] used the empirical formula of Pope [22] to infer information such as number, weight, catch coefficient, and catches for different age groups of chub mackerel. Although there have been some results in stock assessment, there are still large uncertainties in the determination of individual parameters and biological reference points. As suggested by Walters and Maguire [23], even the most abundant and complete data are still challenging for use in formulations and implementations to avoid overfishing.

In recent years, fisheries scientists have paid more and more attention to the lack of data in the stock assessment of fishery populations, and the improvement and development of assessment methods in data-limited situations are also research hotspots of fishery resources at present [24,25,26,27,28,29]. Fish length data are the most readily available data, and length composition or length-frequency (LF) distribution have been used as important information for stock assessment and fisheries management in various assessment models [27,28,30,31]. Moreover, LF data have been used as the main source of data for stock assessments in fisheries where data are lacking [15,25,28,32,33,34,35]. Length-based Bayesian biomass estimation method (LBB) is a new method for population evaluation using LF data only, created by Froese et al. [28], which successfully avoids complex and difficult data such as catch, abundance index, or life history parameters, using a Bayesian Monte Carlo Markov chain method to estimate parameters approximating the development status or relative biomass status of fishery resources through relevant fishery equations. Although the LBB model was developed late, it has been successfully applied to stock assessments of a variety of fish species in several marine areas due to the ease of data availability and comprehensiveness of the results obtained, and it is considered to be a good method for stock assessments in fisheries where data are scarce. For example, Wang et al. [36] used the LBB method to assess seven dominant fish species in the Minjiang Estuary. Wang et al. [17] used the LBB method to assess eight common commercial fish species in the Bohai and Yellow Seas, and the results indicated that the waters were heavily overfished. Richard et al. [37] used the LBB method to assess the stock status of five small pelagic fish species in the Atlantic.

Based on the LBB method, this study analyzed the stock status of chub mackerel in the high seas of the NPO using LF data from 2016 to 2021. The aim was to provide effective scientific support for the formulation of fisheries management strategies by assessing the exploitation and stock status of chub mackerel resources in the high seas of the NPO.

## 2. Materials and Methods

### 2.1. Study Area and Data Resources

**Figure 2:**
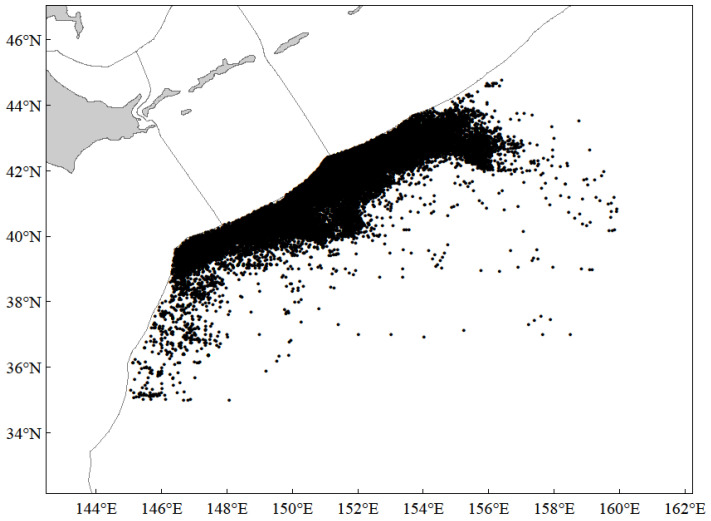
Location distribution (black dots) of chub mackerel catches in the high seas of the NPO from 2016 to 2021.

The study area is located in the high seas of the NPO, covering a fishing ground between 35° N–45° N and 145° E–160° E (Figure 2). The fisheries data of chub mackerel analyzed in this study were obtained from the fishing logs of Chinese commercial light purse seine vessels in the high seas of the NPO from 2016 to 2021, comprising a total of 38,123 fishing operations. Recorded parameters included the start and end time of the operation, the latitude and longitude coordinates of initial net deployment, along with operational net quantities and catches. In this study, 0.5° × 0.5° was defined as the unit fishing area when analyzing the geographical distributions of catches and CPUE. The samples of chub mackerel used in this study came from commercial fisheries, randomly taken and frozen on the spot and then brought back to the lab for measurement, with a total of 4440 samples measured (Table 1). Spearman correlation analysis was performed on chub mackerel CPUE, year, month, longitude, and latitude using SPSS Statistics 19.

### 2.2. Data Analysis

In this paper, the length–weight relationship of the chub mackerel was described by the following formula [38]:

*W* = *aL^b^ * (1)

where *W* is the weight (g) and *L* is the fork length (cm); *a* is the coefficient; *b* is the exponent. The *b* values can reflect the growth status of fish and indicate the unevenness of fish growth, which are caused by the uneven growth of body weight (or volume) and body length [39]. It is generally believed that when the *b* value is approximately 3, the fish will follow isometric growth. When the *b* value is significantly different from 3, the growth of fish will follow an allometric growth pattern. If *b* > 3, fish will show positive allometric growth, and if *b* < 3, fish will show negative allometric growth [40,41].

The Von Bertalanffy growth equation was used to describe the growth status of chub mackerel [42]. The parameters such as *L*_inf_ and *K* were estimated by using the ELEFAN I (electronic length frequency analysis) method in FISAT II software from FAO, and the theoretical initial age *t*_0_ was estimated by using Pauly’s [43] empirical formula. The length group distance was set as 10 mm, and then the fork length and weight growth equation were fitted. The formula was as follows:

*L_t_* = *L*_inf_ [1 − e^−*K*(*t* − *t*^_0_^)^] (2)

log_10_ (−*t*_0_) = −0.3922 − 0.2752log_10_
*L*_inf_ − 1.038 log_10_*K* (3)

where *L_t_* is fork length (cm) of chub mackerel, *L*_inf_ is asymptotic fork length, *K* is the growth coefficient (year)^−1^, and *t*_0_ is the theoretical age at length zero.

The growth performance index (*ϕ*’) was calculated from the following equation [44]:

*ϕ*′ = log_10_*K* + 2log_10_ *L*_inf_ (4)

The mortality of fish is the major factor for the population dynamics, including total mortality (*Z*), natural mortality (*M*), and fishing mortality (*F*). These three can be described as *Z* = *F* + *M*. Total mortality (*Z*) was estimated from the length-converted catch curve using pooled length–frequency data [45] and was calculated by the following equation:

ln(*N_i_ */Δ*t_i_ *) = *c* + *dt_i_ *′ (5)

where *N_i_* is the number of fish in a given length class; Δ*t_i_* is the time needed from the shortest length to the longest length in a given length class for the fish; *t_i_*′ is the age corresponding to length class *i*; *c* and *d* are the intercept and slope of the linear equation, respectively; and *Z* is equal to −*d*.

The natural mortality (*M*) was estimated using the equation by Pauly [45]:

ln*M* = −0.0152 − 0.279ln*L*_inf_ + 0.654ln*K* + 0.463ln*T* (6)

where *T* is the mean temperature of the water layer where fish live; the temperature data obtained from the surveys were 23.61, 22.91, 27.47, 25.38, 25.26, and 25.94 °C, for these six months. The exploitation ratio (*E*) was obtained from *F*/*Z*.

### 2.3. LBB Modeling

In this study, the LBB model was used to estimate the resource status of chub mackerel, and the “TropFishR” package in R 3.6.1 was used for LBB modeling [46]. LBB model is a new method used to estimate the population status using length frequency data only. It is suitable for fish that grow all their lives and reach their maximum length at their maximum age [28]. The LBB model can effectively estimate the asymptotic length (*L*_inf_), length at first capture (*L*_c_), relative natural mortality (*M*/*K*), relative fishing mortality (*F*/*K*), the ratio of the developed biomass of the target species to the undeveloped original biomass (*B*/*B*_0_), and other parameters.

The Von Bertalanffy growth equation (Equation (1)) was used in the LBB model for describing the growth in length. If there is a real and reliable *L*_inf_ actual value, it is recommended to use this value to reduce the uncertainty of the model [36].

After obtaining *L*_inf_, *L*_c_, *M*/*K* and *F*/*K* by LBB model, we use Equation (7) to calculate the length of the maximum unexploited cohort biomass (*L*_opt_) [47]:

(7) $$L_{\mathrm{opt}}=L_{\inf}(\frac{3}{3+M/K})$$

The optimal fork length in first capture (*L*_c-opt_) could be calculated by Equation (8) [48]:

(8) $$L_{c-\mathrm{opt}}=\frac{L_{\inf}(2+3F/M)}{(1+F/M)(3+M/K)}$$

The yield per recruit (*Y*′/*R*) could be calculated by Equation (9) [49]:

(9) $$Y\prime /R\;=\frac{F/M}{1+F/M}{(1\;-L_{c}/L_{\inf})}^{M/K}(1\;-\frac{3(1\;-L_{c}/L_{\inf})}{1+(\frac{1}{M/K\;+\;F/K})}+\frac{3{(1\;-L_{c}/L_{\inf})}^{2}}{1+(\frac{2}{M/K\;+F/K})})+\frac{{(1\;-L_{c}/L\inf)}^{3}}{1+(\frac{3}{M/K\;+F/K})})$$

Assuming that CPUE was proportional to the biomass, *CPUE*′/*R* could be calculated by Equation (10) [49]:

(10) $$\mathrm{CPUE}’/R\;=\frac{Y’/R}{F/M}=\frac{1}{1+F/M}{(1\;-L_{c}/L_{\inf})}^{M/K}(1\;-\frac{3(1\;-L_{c}/L_{\inf})}{1+(\frac{1}{M/K\;+\;F/K})}+\frac{3{(1\;-L_{c}/L_{\inf})}^{2}}{1+(\frac{2}{M/K\;+\;F/K})})+\frac{{(1\;-L_{c}/L_{\inf})}^{3}}{1+(\frac{3}{M/K\;+\;F/K})})$$

The relative biomass of the population without fishing activities (*B*_0_/*R*) could be calculated by Equation (11) [49]:

(11) $${B0\prime /R=(1\;-L_{c}/L_{\inf})}^{M/K}(1\;-\frac{3(1\;-L_{c}/L_{\inf})}{1+(\frac{1}{M/K})}+\frac{3{(1\;-L_{c}/L_{\inf})}^{2}}{1+(\frac{2}{M/K})})+\frac{{(1\;-L_{c}/L_{\inf})}^{3}}{1+(\frac{3}{M/K})})$$

where *B*_0_ is undeveloped biomass. *B*/*B*_0_ could be calculated by using Equation (12) [49]:

(12) $$B/B0=\frac{\mathrm{CPUE}’/R}{B0’/R}$$

When assuming *F* = *M* and *L*_c_ = *L*_c-opt_, the relative biomass of maximum sustainable yields (*B*_msy_/*B*_0_) can be calculated. The current biomass relative to the biomass capable of producing maximum sustainable yields (*B*/*B*_msy_) can be calculated with the following Equation (13):

(13) $$B/B\mathrm{msy}=\frac{B/B0}{B\mathrm{msy}/B0}$$

The prior values and LBB parameters for the six years are shown in Table 2.

## 3. Results

### 3.1. The Spatio-Temporal Distribution of Catches and CPUE of Chub Mackerel

**Figure 3:**
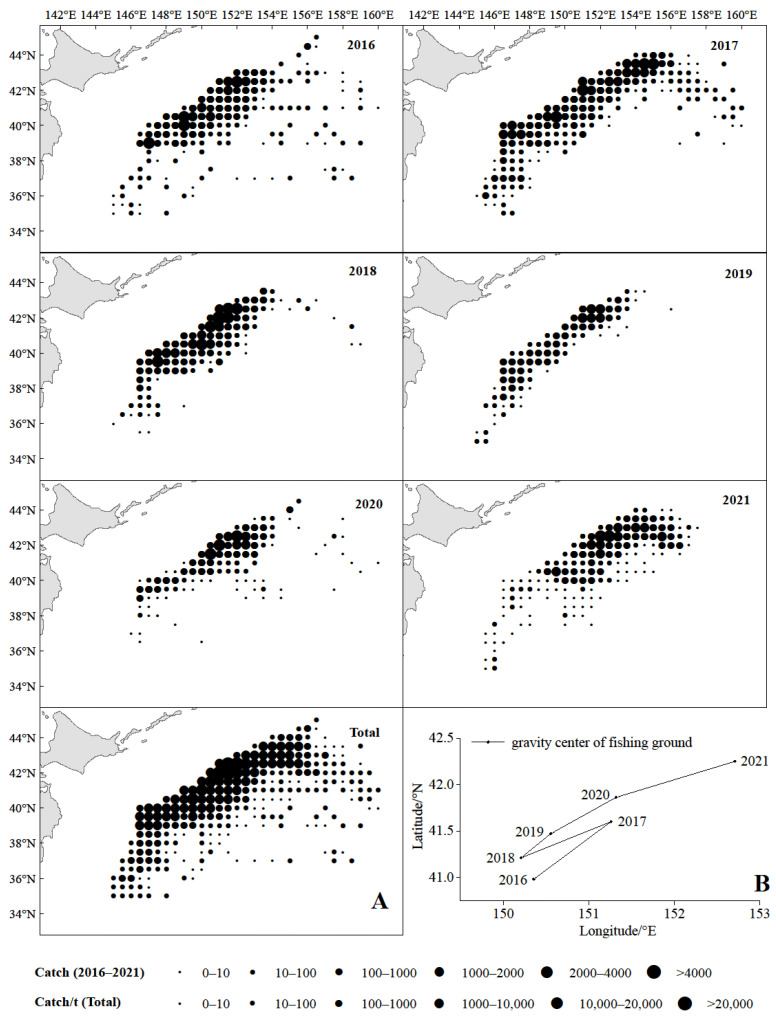
The spatio-temporal dynamics ((**A**), including 2016–2021 and total) and center gravity (**B**) of catches of chub mackerel in the high seas of the NPO from 2016 to 2021.

**Figure 4:**
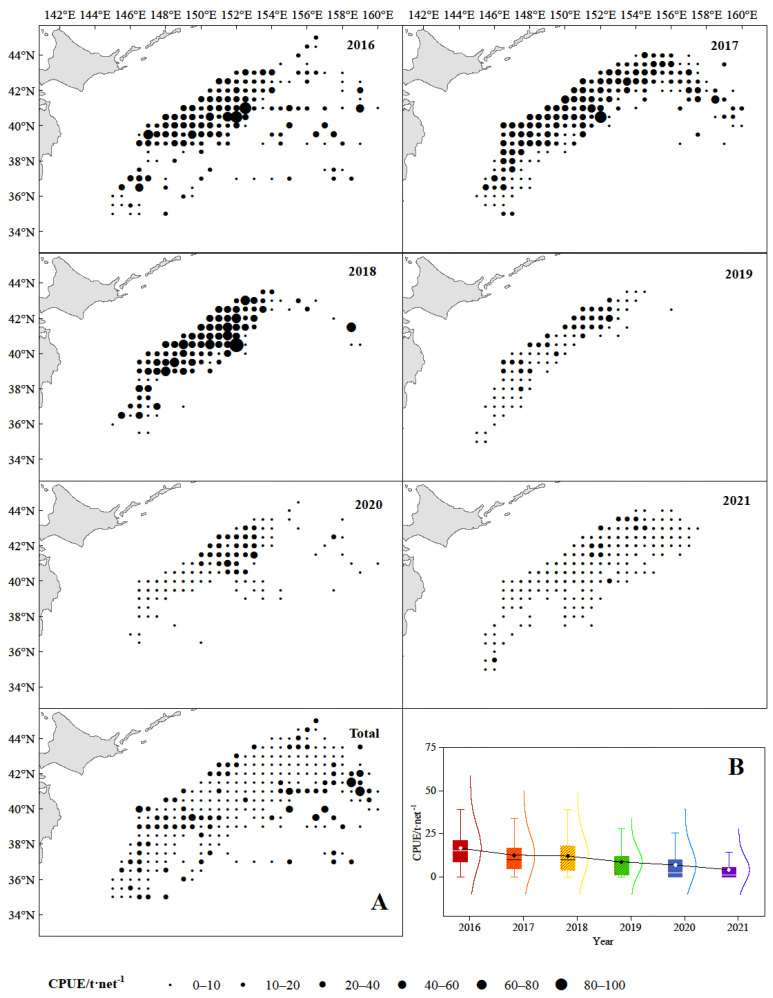
The spatio-temporal dynamics ((**A**), including 2016–2021 and total) and average values (**B**) of CPUE of chub mackerel in the high seas of the NPO from 2016 to 2021.

From 2016 to 2021, fishing activities of chub mackerel were concentrated along the outer edge of Japan and Russia’s exclusive economic zone (EEZ) line, as well as high catches (Figure 3A). The spatial distribution of catches in 2016 was the widest and gradually contracted afterwards. The gravity center of the chub mackerel fishing grounds showed a northeast-southwest-northeast pattern from 2016 to 2021, first moving northwest, then returning in 2018, and then moving northwest continuously (Figure 3B). The CPUE of chub mackerel had a prominent spatio-temporal distribution characteristics, with large values dominating from 2016 to 2018 and small values dominating from 2019 to 2021 (Figure 4A), and the value of CPUE decreased year by year (Figure 4B). The Spearman correlation test showed that there was a strong negative correlation between CPUE and year (r = −0.489, *p* < 0.01) and a weak correlation with month (r = 0.150, *p* < 0.01), longitude (r = −0.098, *p* < 0.01), and latitude (r = −0.012, *p* < 0.01).

### 3.2. Variations in Population Parameters

The basic biological characteristics of chub mackerel, including sex ratio, length range, and mean length, have all shown obvious yearly characteristics (Table 1). The sex ratio (female-to-male) varied between 1.09–2.55 for these six years, which were both greater than 1. The minimum and maximum fork lengths were 102 mm (2021) and 400 mm (2020), respectively. Mean standard length varied from 212.07 mm to 268.28 mm.

The length–weight relationships of chub mackerel from NWPH for these six years were estimated as follows:

2016: *W* = 5.67 × 10^−7^*L*^3.53^ (*R*^2^ = 0.9985, n = 315, *p* < 0.001)

2017: *W* = 1.02 × 10^−7^*L*^2.98^ (*R*^2^ = 0.9972, n = 934, *p* < 0.001)

2018: *W* = 6.05 × 10^−7^*L*^2.84^ (*R*^2^ = 0.9941, n = 294, *p* < 0.001)

2019: *W* = 2.67 × 10^−7^*L*^3.00^ (*R*^2^ = 0.9974, n = 1236, *p* < 0.001)

2020: *W* = 1.07 × 10^−7^*L*^3.19^ (*R*^2^ = 0.9963, n = 868, *p* < 0.001)

2021: *W* = 2.45 × 10^−5^*L*^3.02^ (*R*^2^ = 0.9873, n = 1234, *p* < 0.001)

**Figure 5:**
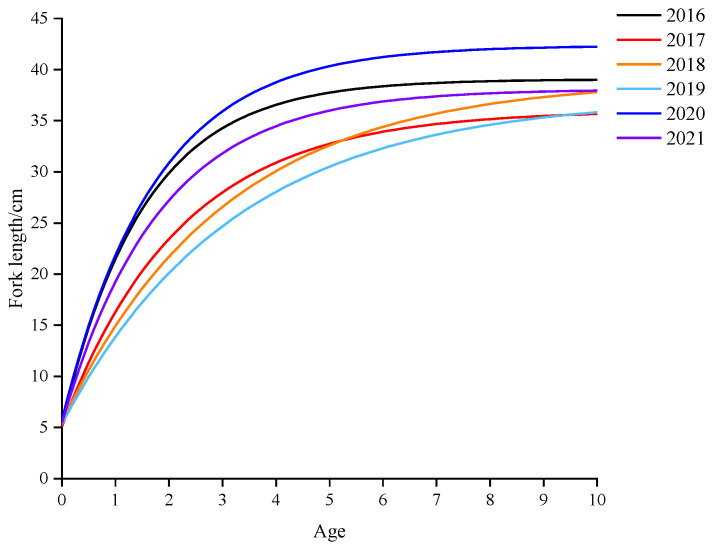
The growth curves of fork length in Von Bertalanffy functions of chub mackerel in the high seas of the NPO from 2016 to 2021.

The growth curve of chub mackerel is shown in Figure 5, and the Von Bertalanffy growth functions for these six years calculated by ELEFAN I were as follows:

2016: *L_t_* = 39.05 × (1 − e^−0.65 × (*t* + 0.23)^)

2017: *L_t_* = 36.00 × (1 − e^−0.45 × (*t* + 0.34)^)

2018: *L_t_* = 39.03 × (1 − e^−0.33 × (*t* + 0.46)^)

2019: *L_t_* = 37.28 × (1 − e^−0.31 × (*t* + 0.50)^)

2020: *L_t_* = 42.35 × (1 − e^−0.58 × (*t* + 0.25)^)

2021: *L_t_* = 38.08 × (1 − e^−0.55 × (*t* + 0.28)^)

For length–weight relationships over these six years, the range of *a* value was from 1.02 × 10^−7^ in 2017 to 3.65 × 10^−5^ in 2021, and *b* value was from 2.78 in 2021 to 3.19 in 2018 (Table 3). The extreme length (*L*_inf_) varied from 36 cm to 42.35 cm. The growth coefficient *K* varied from 0.31 to 0.65, and no substantial difference occurred in the growth performance index *ϕ*’ over time. The total mortality coefficient *Z* changed with the years, ranging from 1.01 in 2016 to 1.64 in 2021. The natural mortality coefficient *M* and the fishing mortality coefficient *F* varied between 0.59 and 0.85 and 0.23 and 0.79, respectively. The exploration rate *E* was lower than 0.5 in all the years (Table 3).

### 3.3. Exploitation Status of Chub Mackerel

**Figure 6:**
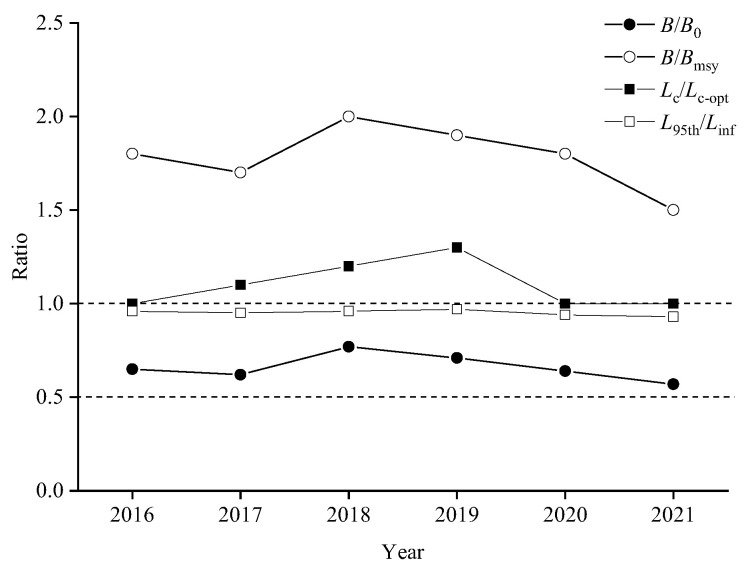
The exploitation status of chub mackerel from 2016 to 2021.

**Figure 7:**
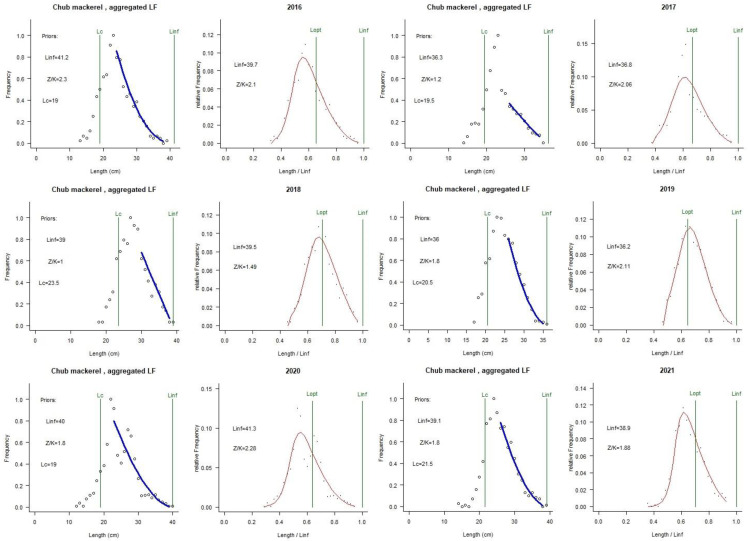
Assessment results of the length-based Bayesian (LBB) method for chub mackerel.

Figure 6 and Table 4 show the stock assessment results for chub mackerel using the LBB method. The asymptotic fork length of chub mackerel varied from 36.2 cm (2019) to 41.3 cm (2020). No overfishing occurred in the population of this species during the period 2016–2021. The parameters *F*/*M* were all less than 0.5, indicating that the fishing mortality was less than natural mortality. The *E* values ranged from 0.17 to 0.32, all of which were below 0.5, suggesting that fishing pressure was unlikely to be the primary driver of the decline in the chub mackerel population. The parameters *B*/*B*_0_ were greater than 0.5 and *B*/*B*_msy_ were greater than 1 in 2016–2021, indicating that the chub mackerel resource is at a high level. The parameters *L*_95th_/*L*_inf_ and *L*_mean_/*L*_opt_ were close to 1, while the parameters *L*_c_/*L*_opt_ were greater than 1, which proved that the body length structure of chub mackerel was reasonable and large individuals were still present (Figure 7, Table 4).

## 4. Discussion

### 4.1. Analysis of Spatio-Temporal Distribution of Catches and CPUE

Chub mackerel is the main species in the commercial light purse seine fisheries in the high seas of the NPO, but both the catch and the proportion of catches have shown a downward trend year by year [50], as well as the CPUE (Figure 3B). However, our assessment model in this study also reached the conclusion that the chub mackerel has not been overexploited and its population status is relatively healthy (Figure 4). It is reported that the chub mackerel in the high seas of the NPO has certain development potential [20]. In terms of this problem, we speculate that there may be the following three reasons: the first is that although the biomass of chub mackerel have decreased, which is a reasonable resource fluctuation, it has not reached the critical value that affects the normal growth of the chub mackerel population. Second, the spawning grounds of the chub mackerel in the middle and south of the Pacific coast of Japan [51,52] may have changed, resulting in a decrease in the number of recruitment groups transported to the nursery area in the Pacific waters off northeast Japan by the Kuroshio Current [1], which has in turn led to a decrease in the number of mature individuals caught by commercial vessels. The high seas of the NPO are the main feeding grounds and high-suitability areas of chub mackerel [53], and the changes in environmental factors there have not been unfavorable to their growth. Consequently, individuals caught in commercial fisheries did not show significant miniaturization in body size, which leads to the conclusion that assessment models based on body length indicate that the chub mackerel stock still has potential for exploitation [20]. Third, the competition for available food is increasing. Studies have shown that the catch and catch proportion of Japanese sardine (*Sardinops melanostictus*) in the high seas of the NPO have shown an increasing trend year by year [50], leading to increased food competition with chub mackerel larvae groups. However, although the adult chub mackerel individuals compete with Japanese sardine, the latter is also a major food source for chub mackerel [54]. Although the number of adult chub mackerel individuals has decreased, the body length has not changed significantly due to these two reasons. It is worth noting that our results show that the *E* value shows a trend of increasing year by year, reaching 0.48 in 2021 (Table 3). Although there is no over-exploitation, it may soon reach the critical value. Attention should be paid to this by international management organizations.

This study found that year and month significantly affected the CPUE of chub mackerel, indicating significant interannual and seasonal variations in its abundance [8,55,56]. We also found that large CPUE values dominated from 2016 to 2018, while small values prevailed from 2019 to 2021 (Figure 3A). CPUE showed a year-on-year decline during this period (Figure 3B). Interestingly, as shown in Figure 1, the catches of chub mackerel from 2016 to 2018 were significantly higher than those from 2019 to 2021. Zhao et al. [8] argued that high catches in the high seas of the NPO are closely associated with high fishing effort, which may have contributed to the sharp decline in catches in 2019. However, the number of fishing vessels in 2021 was the peak in the previous years, but catches have not yet returned to the levels of 2017 and 2018. Han et al. [57] noted that there were three main reasons for this phenomenon: the first is the primary contributing factors of the proliferation of fishing vessels and the resulting interference among their fish-attracting light systems [58]; the second is that the interspecific food competition and density effects between Japanese sardine and chub mackerel [59] have led to a decline in the chub mackerel stock; third, the escalating catch volumes of Japanese sardine in recent years have resulted in fishing pressure levels that exceed the adaptive capacity of chub mackerel [57].

### 4.2. Analysis of Gravity Center of Fishing Grounds

In general, the gravity center of chub mackerel fishing grounds showed a trend moving towards the northeast (Figure 3B), which has been observed since 1970 [11]. In terms of this phenomenon, climate warming is considered to be the most important factor. Temperature is the most important environmental variable affecting the dynamics and spatial distribution of marine fish populations [60,61]. The distribution range of most marine species changes along the isotherm to higher latitudes (i.e., to the polar direction) or to deeper waters, but complex hydrological conditions may also cause species to move in other directions [60,62,63,64]. For a long time, the sea surface temperature (SST) in the NPO has played an important role in the rate of climate change. Since 2014, frequent marine heatwave events have occurred in the NPO [65], leading to significant changes in the distribution of marine species [66], the most obvious phenomenon of which is the gradual northward shift of the fishing grounds in the high seas of the NPO. It is reported that Japanese sardine have been observed crossing the Pacific Ocean to the California Current Large Marine Ecosystem of the eastern Pacific due to the increases in frequency and intensity of warm water anomalies and marine heatwaves, which has never happened before [66]. The SST ranges for chub mackerel habitats are 7–19 °C in spring (optimal: 11–15 °C) and 8–24 °C in summer (optimal: 8–12 °C) [67]. Xu et al. [56] suggested an optimal SST range of 13–20.18 °C. A northward habitat shift has been observed with increasing SST [8]. Zhuang et al. [68] reported concentrated feeding migrations during August to October, coinciding with biological peak conditions in September to October when temperatures optimally support growth in the high seas of the NPO. Therefore, in the context of climate warming, how to maintain the balance of marine biological population structure in marine ecosystems is a great challenge for management organizations.

### 4.3. Stock Assessment Analysis

The LBB model offers a new approach to stock assessment in data-poor fisheries, which requires only enough length frequency (LF) data to obtain a more comprehensive set of resource parameters and is more inclusive for fisheries with unreliable catch data or lacking the necessary data [37]. The LBB model requires that the length data must be representative, and if the LF cannot represent the composition of the exploited population, or if the LF is affected by the supplemental population under the interaction of growth rate or mortality, the results produced by the LBB will have a large error [28]. This model is suitable for species that grow throughout their lives, such as commercially important fish and invertebrates, and for which the maximum body length is also representative of the maximum age, that is, the body length structure can reflect the true age composition [28]. It also puts forward a new indicator of whether the structure of resource development groups is sufficient [28]. The fishing grounds of chub mackerel in the high seas of the NPO are far away from the mainland, where scientific surveys or long-term data collection are challenging, and applying limited data to models that require complete data will make the assessment results questionable [69,70,71]. Since the LBB model was proposed, it has been successfully applied to fisheries stock assessment and proven to be robust [17,20,37,72].

According to the stock assessment results of the LBB model, the ratios of *L*_9_5th/*L*_inf_ and *L*_mean_/*L*_opt_ were close to 1, while the parameters *L*_c_/*L*_opt_ were both greater than 1, which proved that there was no miniaturization in the length, large individuals were still present, and the population resources were in good condition. By comparing the values of *M*/*K*, *F*/*K*, and *Z*/*K*, we could find that the total mortality was mainly contributed to by natural mortality, rather than fishing mortality, indicating that the current fishery in the high seas of the NPO had little impact on chub mackerel resources. Yakami et al. [6] pointed out that the Pacific group of chub mackerel in NPO still had a great development potential. However, it must be mentioned that although the LBB model had been widely used, its accuracy had been questioned. Hordyk et al. [73] stated that the master equation of LBB was incomplete and cannot correct the pile-up effect caused by aggregating length data into the same length group. Froese et al. [28] responded by stating that the simulated data from the reaction model assumptions would always be more suitable for the model than the data generated from deviations from the assumptions, but the growth and death of fish in the real world are uncertain, and the premise of the accumulation effect in Hordyk et al. [73] is biased and too idealistic for the operation of most models. Also, Froese et al. [74] indicated that the premise assumptions of Hordyk et al. [73] regarding the stacking effectwere somewhat biased and too idealistic for most of the models to operate. Length-based models tend to be highly sensitive to the assumed value of *L*_inf_, while LBB models may be less sensitive to estimating *L*_inf_ due to limited simulation testing [27]. Froese et al. [72] stated that if users can obtain an accurate *L*_inf_ estimates through independent studies, users can introduce the value autonomously.

In fisheries stock assessment, the sensitivity to sample size significantly impacts the reliability and uncertainty of the assessment results [75]. The simulation results of Zhang et al. [75] demonstrate that the parameters estimated by the LBB method are insensitive to sample sizes exceeding 100. When the sample size is insufficient, it will lead to the following consequences: first, when the sample size is too small, the length-frequency distribution cannot accurately reflect the true population structure, causing particularly significant deviations in the growth parameter *L*_inf_ within age groups exhibiting multimodal distributions [27]. The second is that the estimation of mortality rate is unstable. The absence of large individuals in small samples can lead to an overestimation of natural mortality rate, at which point the Z/M ratio may be underestimated [27]. Third, a small sample size can lead to increased uncertainty in biomass estimation [28,75,76].

In general, the LBB model provides a convenient stock assessment method for those fishery populations that are difficult to obtain reliable catch data or biological data for.

## 5. Conclusions

The aim of this study is to analyse the spatio-temporal distribution dynamics of fishing grounds and the population status of chub mackerel in the high seas of the NPO, which is useful to fishery management. The results demonstrated the following: (1) The gravity center of the fishing grounds of chub mackerel moved northward, and the CPUE decreased year by year. (2) From 2016 to 2021, chub mackerel in the high seas of the NPO grew well, without miniaturisation and with a reasonable body length structure and a certain number of large individual groups. (3) The fishing mortality rate was low, the variation in the resource of chub mackerel was not affected by fishing factors, and there was no overfishing. (4) Although there is no over-exploitation, it may soon reach the critical value due to the increase in the *E* value. Also, we suggest that managers should focus on the impact of climate warming on the population composition of marine ecosystems.

## Figures and Tables

**Table 1:** Samples of chub mackerel.

Year	Numbers	Sex Ratio (**♀**/**♂**)	Length Range/mm	Mean Length/mm
2016	315	1.56	129~389	212.07
2017	934	1.09	130~345	223.15
2018	294	1.43	175~388	268.28
2019	868	1.38	165~358	240.18
2020	1234	1.61	110~400	237.08
2021	795	2.55	102~385	246.17

**Table 2:** Scenarios and prior values of chub mackerel.

Year	Class Bin (mm)	*L*_inf_ prior	*Z*/*K* prior	*M*/*K* prior	*F*/*K* prior	*L_c_* prior	*Alpha* prior
2016	10	41.2	2.29	1.5	0.78	19.4	29.9
2017	10	36.3	1.22	1.5	0.3	19.9	17.9
2018	10	39	1.03	1.5	0.3	24	19.6
2019	10	36	1.77	1.5	0.27	20.9	19.7
2020	10	40	1.76	1.5	0.26	19.4	29.5
2021	10	39.1	1.78	1.5	0.28	21.9	28.7

**Table 3:** Variations in growth and mortality parameters of chub mackerel.

Year	2016	2017	2018	2019	2020	2021
*a*	5.67 × 10^−7^	1.02 × 10^−7^	1.62 × 10^−7^	1.12 × 10^−7^	8.43 × 10^−7^	3.65 × 10^−5^
*b*	3.53	3.43	3.75	3.39	3.46	2.78
*L*_inf_	39.05	36.02	39.01	36.30	42.35	38.08
*K*	0.65	0.75	0.88	0.42	0.58	0.55
*t_0_*	−0.23	−0.15	−0.17	−0.37	−0.25	−0.28
*ϕ*′	2.99	2.98	2.70	2.74	2.99	2.90
*Z*	1.01	1.80	1.81	1.25	1.25	1.64
*M*	0.78	1.15	1.24	0.78	0.71	0.85
*F*	0.23	0.65	0.57	0.47	0.55	0.79
*E*	0.23	0.36	0.31	0.37	0.44	0.48

**Table 4:** Summary of the length-based Bayesian (LBB) results.

Year	2016	2017	2018	2019	2020	2021
*L*_inf_	39.7	36.8	39.5	36.2	41.3	38.9
*L*_opt_	26	25	28	23	26	27
*L_c-_*_opt_	19	19	20	17	20	21
*M*/*K*	1.58	1.48	1.24	1.64	1.71	1.27
*F*/*K*	0.52	0.58	0.26	0.47	0.57	0.61
*Z*/*K*	2.1	2.06	1.5	2.1	2.32	1.89
*F*/*M*	0.33	0.39	0.21	0.29	0.34	0.46
*E*	0.24	0.26	0.17	0.22	0.25	0.32
*B*/*B*_0_	0.65	0.62	0.77	0.71	0.64	0.57
*B*/*B*_msy_	1.8	1.7	2	1.9	1.8	1.5
*L*_c_	19.8	20.5	24.4	22.4	20.4	22
*L_mean_*/*L_c-_*_opt_	0.97	1	1	1.1	0.98	0.97
*L*_c_/*L_c-_*_opt_	1	1.1	1.2	1.3	1	1
*L*_95th_/*L_inf_*	0.96	0.95	0.96	0.97	0.94	0.93
Status	healthy	healthy	healthy	healthy	healthy	healthy

## Data Availability

The data presented in this study are available on request from the corresponding author. The data are not publicly available due to privacy restrictions.
